# Draft genome sequences of 12 *Agrobacterium* strains isolated from coastal plants in Taiwan

**DOI:** 10.1128/mra.01046-25

**Published:** 2025-11-05

**Authors:** Hsi-Ching Yen, Fan-Chen Huang, Pei-Ru Chien, Chih-Lin Wu, Liang-Yu Chen, Chuan-Wen Ho, Guo-Zhang M. Song, Wei-Jen Lin, Hsing-Juh Lin, Chieh-Chen Huang, Hau-Hsuan Hwang, Chih-Horng Kuo

**Affiliations:** 1 Institute of Plant and Microbial Biology, Academia Sinica 71559, Taipei, Taiwan; 2 GeneReach Biotechnology Corp 694968 , Taichung, Taiwan; 3 Department of Life Sciences, National Chung Hsing University 593984, Taichung, Taiwan; 4 Department of Soil and Water Conservation, National Chung Hsing University 594204, Taichung, Taiwan; 5 Department of Biological Resources, National Chiayi University 63107, Chiayi, Taiwan; 6 Innovation and Development Center of Sustainable Agriculture, National Chung Hsing University 34916, Taichung, Taiwan; 7 Advanced Plant and Food Crop Biotechnology Center, National Chung Hsing University 34916, Taichung, Taiwan; 8 Biotechnology Center, National Chung Hsing University 34916, Taichung, Taiwan; University of Manitoba, Winnipeg, Canada

**Keywords:** genome, *Agrobacterium*, endophyte, Taiwan

## Abstract

We report draft genome sequences of 12 *Agrobacterium* strains isolated from coastal plants in Taiwan. Assemblies range from 4.95 to 5.42 Mb with >97.5% completeness. Genomic analyses reveal species identities and secretion system genes, providing resources for future research.

## ANNOUNCEMENT

The genus *Agrobacterium* comprises diverse species associated with soil and plants. Some strains harbor oncogenic plasmids and can be used for *Agrobacterium*-mediated transformation (AMT) (1). A major limitation of AMT is that many plants are recalcitrant to common laboratory strains. Early work demonstrated that wild-type *Agrobacterium* strains differ substantially in host range, suggesting that strain diversity could broaden AMT application to different crops (2). Guided by this rationale, we screened draft genome assemblies from our collection of endophytic bacteria isolated from coastal plants in Taiwan, identified 12 strains as members of the genus *Agrobacterium*, and report their genome sequences in this work (Table 1).

**TABLE 1:** Metadata and genome assembly statistics of the strains included in this study^*a*^

Strain	Species	Similarity to type strain (AF/ANI %)	Collection date	Location	GPS coordinates	Host	SRA accession	Assembly accession	Number of reads	Coverage (fold)	Number of contigs	Contig N50 (bp)	Assembly size (bp)	GC content (%)	Number of annotated genes	Completeness (%)	Contamination (%)	T6SS genes
NCHU4840	*Agrobacterium cavarae*	85.5/96.2	02-03-2022	Taiwan: Miaoli County, Tongxiao Township	24.495968 N 120.671137 E	*Bidens pilosa*	SRR34682073	GCA_051590745.1	5,511,444	159.6	27	422,750	5,048,636	58.2	4,644	97.72	0.68	Complete
NCHU4860	*Agrobacterium cavarae*	84.7/96.2	02-03-2022	Taiwan: Miaoli County, Tongxiao Township	24.495968 N 120.671137 E	*Urena lobata*	SRR34681896	GCA_052261035.1	6,259,710	183.5	15	884,046	4,951,969	58.2	4,579	97.76	0.95	Complete
NCHU4975	*Agrobacterium cavarae*	85.7/96.2	02-03-2022	Taiwan: Miaoli County, Tongxiao Township	24.495968 N 120.671137 E	*Ipomoea pes-caprae* subsp. *brasiliensis*	SRR34682131	GCA_052261055.1	5,653,282	162.8	29	403,161	5,048,170	58.2	4,647	97.72	0.68	Complete
NCHU4981	*Agrobacterium pusense*	89.3/98.2	02-03-2022	Taiwan: Miaoli County, Tongxiao Township	24.495968 N 120.671137 E	*Ipomoea pes-caprae* subsp. *brasiliensis*	SRR34692460	GCA_052261075.1	6,124,078	166.2	37	654,217	5,355,332	59.1	5,079	99.60	0.77	Absent
NCHU5031	*Agrobacterium pusense*	88.6/98.2	09-05-2022	Taiwan: Miaoli County, Zhunan Township	24.675569 N 120.830987 E	*Oenothera laciniata*	SRR34685741	GCA_052261095.1	7,600,106	206.6	23	1,004,322	5,409,650	59.1	5,115	99.33	0.95	Absent
NCHU5032	*Agrobacterium pusense*	87.7/97.6	09-05-2022	Taiwan: Miaoli County, Zhunan Township	24.675569 N 120.830987 E	*Oenothera laciniata*	SRR34687319	GCA_052261115.1	6,567,210	178.4	30	440,007	5,416,145	59.3	5,172	99.55	0.62	Partial
NCHU5334	*Agrobacterium salinitolerans*	89.2/99.2	04-06-2022	Taiwan: Tainan City, Qigu District	23.157924 N 120.077311 E	*Bidens pilosa*	SRR34682062	GCA_052261135.1	6,247,712	175.8	29	443,794	5,207,595	59.3	4,955	99.71	0.29	Complete
NCHU5640	*Agrobacterium cavarae*	85.7/96.2	22-08-2022	Taiwan: Yilan County, Toucheng Township	24.913217 N 121.877840 E	*Murdannia keisak*	SRR34682063	GCA_052261155.1	5,375,518	155.8	43	405,051	5,006,747	58.2	4,677	97.52	1.30	Complete
NCHU5641	*Agrobacterium cavarae*	85.4/96.2	22-08-2022	Taiwan: Yilan County, Toucheng Township	24.913217 N 121.877840 E	*Murdannia keisak*	SRR34685756	GCA_052261175.1	6,621,420	193.0	43	405,051	5,006,273	58.2	4,680	97.52	1.30	Complete
NCHU5644	*Agrobacterium cavarae*	85.6/96.2	22-08-2022	Taiwan: Yilan County, Toucheng Township	24.913217 N 121.877840 E	*Murdannia keisak*	SRR34692412	GCA_052261195.1	5,096,434	147.9	41	421,673	5,006,542	58.2	4,676	97.79	1.30	Complete
NCHU5645	*Agrobacterium cavarae*	85.2/96.2	22-08-2022	Taiwan: Yilan County, Toucheng Township	24.913217 N 121.877840 E	*Ipomoea pes-caprae* subsp. *brasiliensis*	SRR34685760	GCA_052261215.1	5,920,386	170.7	44	405,051	5,006,869	58.2	4,671	97.52	1.30	Complete
NCHU5738	*Agrobacterium deltaense*	92.0/97.9	14-10-2022	Taiwan: Changhua County, Fangyuan Township	23.928038 N 120.314438 E	*Pluchea indica*	SRR34685761	GCA_052261235.1	5,704,246	161.2	41	350,740	5,158,671	59.9	4,914	99.47	1.70	Complete

^*a*^ AF, alignment fraction; ANI, average nucleotide identity; T6SS, type VI secretion system.

Unless otherwise noted, methods followed the cited references, kits were used according to the manufacturers’ instructions, and bioinformatics tools were used with default settings. For strain isolation, root samples were surface-sterilized, homogenized in distilled water, serially diluted, spread onto Luria Broth (LB) agar, and incubated at 30°C for 48 h to obtain single colonies, as described (3). Colonies were re-streaked at least three times to ensure purity. For DNA extraction, a single colony was cultured in 1 mL LB broth at 30°C for 24 h, then processed sequentially using the Wizard Genomic DNA Purification Kit (Promega) and DNeasy Blood and Tissue Kit (Qiagen).

For shotgun sequencing, libraries were prepared using the SATLite DNA Prep Kit with the SATLite 2.0 Library Prep Automation Device (GeneReach Biotechnology) and sequenced on a NovaSeq X Plus using the 10B Reagent Kit (Illumina) to obtain paired-end 150 bp reads. Raw reads were trimmed using fastp v1.0.1 (4) and assembled using SPAdes v3.15.0 (5) with the “--isolate” option (k  =  21, 33, 55, and 77). Contigs >500 bp were retained. Trimmed reads were mapped to contigs using BWA v0.7.17-r1188 (6), and coverage was assessed using SAMtools v1.9 (7). For species identification, assemblies were compared against NCBI RefSeq genomes (8) with FastANI v1.33 (9). Assemblies were submitted to GenBank and annotated using the NCBI Prokaryotic Genome Annotation Pipeline v6.10 (10). Completeness and contamination were estimated using CheckM v1.2.4 (11). MacSyFinder v2.1.4 (12) was used to identify genes encoding type IV and type VI secretion systems, which are relevant to *Agrobacterium* virulence and ecology, respectively (13). To identify contigs corresponding to oncogenic plasmids, coding sequences from reference plasmids (14, 15) were queried using BLASTP v2.11.0 (16) against annotated coding sequences.

Results are summarized in Table 1. All strains showed >84% alignment fraction and >96% average nucleotide identity to their respective type strains, allowing confident species assignment (17). Contig counts ranged from 15 to 44, N50 from 351 to 1,004 kb, assembly sizes from 4.95 to 5.42 Mb, GC content from 58.2 to 59.9%, and annotated genes from 4,579 to 5,172. All assemblies showed >97.5% completeness and <2% contamination. No oncogenic plasmid contigs nor type IV secretion system genes were detected. The feasibility of introducing oncogenic plasmids into these strains by conjugation remains to be tested. These genome assemblies provide useful references for future studies of these strains and comparative genomics of *Agrobacterium*.

## Data Availability

The raw sequencing reads and draft genome assemblies have been deposited in NCBI GenBank and are publicly available; accession numbers are listed in Table 1.
